# Superoxide Production by the Red Tide-Producing *Chattonella marina* Complex (Raphidophyceae) Correlates with Toxicity to Aquacultured Fishes

**DOI:** 10.3390/antiox10101635

**Published:** 2021-10-17

**Authors:** Tomoyuki Shikata, Koki Yuasa, Saho Kitatsuji, Setsuko Sakamoto, Kazuki Akita, Yuichiro Fujinami, Yoshitaka Nishiyama, Toshihisa Kotake, Ryusuke Tanaka, Yasuhiro Yamasaki

**Affiliations:** 1 Fisheries Technology Institute, Japan Fisheries Research and Education Agency, 122-7 Nunoura, Tamanoura-cho, Goto, Nagasaki 853-0508, Japan; akitak@affrc.go.jp (K.A.); fujinami@affrc.go.jp (Y.F.); 2 Fisheries Technology Institute, Japan Fisheries Research and Education Agency, 2-17-5 Maruishi, Hatsukaichi, Hiroshima 739-0452, Japan; yuasa_koki98@fra.go.jp (K.Y.); sahok@affrc.go.jp (S.K.); sssaka@affrc.go.jp (S.S.); 3 Department of Biochemistry and Molecular Biology, Graduate School of Science and Engineering, Saitama University, 255 Shimo-Okubo, Sakura-ku, Saitama 338-8570, Japan; nishiyama@mail.saitama-u.ac.jp (Y.N.); kotake@mail.saitama-u.ac.jp (T.K.); 4 Department of Marine Biology and Environmental Sciences, Faculty of Agriculture, University of Miyazaki, Miyazaki 889-2192, Japan; rtanaka@cc.miyazaki-u.ac.jp; 5 Department of Applied Aquabiology, National Fisheries University, 2-7-1, Nagata-Honmachi, Shimonoseki, Yamaguchi 759-6595, Japan; yamasaky@fish-u.ac.jp

**Keywords:** harmful algal bloom, interstrain variation, oxidant stress, reactive oxygen species (ROS)

## Abstract

The marine raphidophyte *Chattonella marina* complex forms red tides, causing heavy mortalities of aquacultured fishes in temperate coastal waters worldwide. The mechanism for *Chattonella* fish mortality remains unresolved. Although several toxic chemicals have been proposed as responsible for fish mortality, the cause is still unclear. In this study, we performed toxicity bioassays with red sea bream and yellowtail. We also measured biological parameters potentially related to ichthyotoxicity, such as cell size, superoxide (O_2_^•−^) production, and compositions of fatty acids and sugars, in up to eight *Chattonella* strains to investigate possible correlations with toxicity. There were significant differences in moribundity rates of fish and in all biological parameters among strains. One strain displayed no ichthyotoxicity even at high cell densities. Strains were categorized into three groups based on cell length, but this classification did not significantly correlate with ichthyotoxicity. O_2_^•−^ production differed by a factor of more than 13 between strains at the late exponential growth phase. O_2_^•−^ production was significantly correlated with ichthyotoxicity. Differences in fatty acid and sugar contents were not related to ichthyotoxicity. Our study supports the hypothesis that superoxide can directly or indirectly play an important role in the *Chattonella*-related mortality of aquacultured fishes.

## 1. Introduction

When microalgae grow largely to change the water color in coastal areas, we call the phenomenon “red tide”. A part of red tides adversely affects fisheries and tourism [1]. The *Chattonella marina* complex (hereafter referred to as *Chattonella*) is a marine raphidophyte that forms red tides, causing tremendous mortality of aquacultured organisms, mainly fish, in temperate coastal waters around the world [2,3]. This raphidophyte includes *Chattonella antiqua*, *C. marina*, *C. ovata*, and *C. minima*, which were formerly distinguished by morphological features [4,5,6,7,8].

In order to develop specific mortality mitigation techniques, it is necessary to determine the mechanism for the mortality of aquacultured fish by *Chattonella*. Studies over about 40 years have revealed that decreases in the blood oxygen level and osmotic injury following lesions and the clogging of gills caused by *Chattonella* cells could be lethal for fish, but the toxic chemicals responsible for these effects are still unknown [9]. The long history of these studies has revealed the difficulties and highlighted areas for more focused efforts [10].

The maintenance of aquacultured fishes for bioassays of *Chattonella* toxicity requires much effort. As an alternative, small-scale bioassays using fish tissues such as branchial cells, zooplankton, and animal erythrocytes have been used. However, the results from small-scale bioassays often contradict those from bioassays using whole fish, suggesting that small-scale bioassays risk yielding misleading results regarding the mechanisms of mortality. It is known that only live *Chattonella* cells can kill aquacultured fishes such as red sea bream (*Pagrus major*) and yellowtail (*Seriola quinqueradiata*); ruptured cells and culture supernatant have no toxicity to whole fish [11,12]. Marine medaka exposed to ruptured cells, supernatant, and organic extracts from *Chattonella* cells can also survive [13]. On the other hand, the mortality of rainbow trout gill cells exposed to ruptured *Chattonella* cells is much higher than for those exposed to intact cells [14,15]. Rotifers also show high mortality when exposed to organic extracts from *Chattonella* cells [16]. There is considerable hemolysis upon exposure to organic extracts from *Chattonella* cells [17,18], but no significant hemolytic activity is detected in either cell suspension or cell-free culture supernatant [19]. These results suggest the possibility that the mechanisms of mortality differ between whole fish and the other smaller organisms or tissues.

Precise control of *Chattonella* culture is also key to accurate toxic assessment because the toxicity of *Chattonella* is considerably influenced by its physiological state [10,13]. *Chattonella* fails to achieve stable growth without frequent culture transfer, careful control of environmental conditions such as temperature and light intensity, and the selection of clean seawater as the basis for the culture medium. This is magnified with larger-scale culture because of a decrease in growth rate and maximum yield, or no growth, depending on the strain cultured. Because of these challenges, there has been little progress in identifying the compounds responsible for mortality, but several candidates have been proposed.

Natural red-tide seawater and some cultured strains of *Chattonella* contain neurotoxins such as brevetoxin-like compounds [20,21,22], but other cultured strains with strong ichthyotoxicity contain low levels or no neurotoxins [23], suggesting that this type of toxin is not the main compound responsible for fish mortality. Reactive oxygen species (ROS) such as superoxide (O_2_^•−^) and hydrogen peroxide have been recognized as chemicals responsible for branchial lesions and mucus secretion in fish [12]. *Chattonella* cells produce and secrete large amounts of ROS extracellularly in comparison with other microalgae [15,24,25,26]. *Chattonella* can produce O_2_^•−^ via NADPH oxidase in the cell membrane using intracellular reduction power from photosynthesis and so on [27]. However, it is unclear how *Chattonella* defends from high levels of O_2_^•−^, although the research on the mechanisms for quenching intracellular ROS has advanced lately [28]. Some studies have suggested that fatty acids (FAs) and those oxidized by ROS from *Chattonella* were associated with branchial lesions [14,23,29]. *Chattonella* cells have a structural coating, namely glycocalyx, composed of acid mucopolysaccharides, which may be related to their high viscosity [30,31]. Microhistological analyses using the indirect immunofluorescent antibody technique have visualized the glycocalyx from *Chattonella* cells attached to fish gills [32].

These findings have been accumulated using a variety of *Chattonella* strains, experimental and analytical conditions, and toxic assessment systems, including small-scale bioassays. These findings should therefore be verified by using a single experimental design to accurately narrow down the parameters responsible for the mortality of aquacultured fish. The toxicity of *Chattonella* may depend on culture strain [12], although it is unclear if there are significant differences in toxicity among the former three species classified by morphology, such as cell size [33]. A comparative study using strains with different ichthyotoxicity could be effective for identifying the toxic parameter(s) of *Chattonella*. Therefore, we used eight strains of *Chattonella* to conduct toxic bioassays with red sea bream and yellowtail. We measured *Chattonella* cell size, O_2_^•−^ production, and FA and sugar content, and then statistically extracted parameters correlated with ichthyotoxicity.

## 2. Materials and Methods

### 2.1. Chattonella Strains

**Figure 1:**
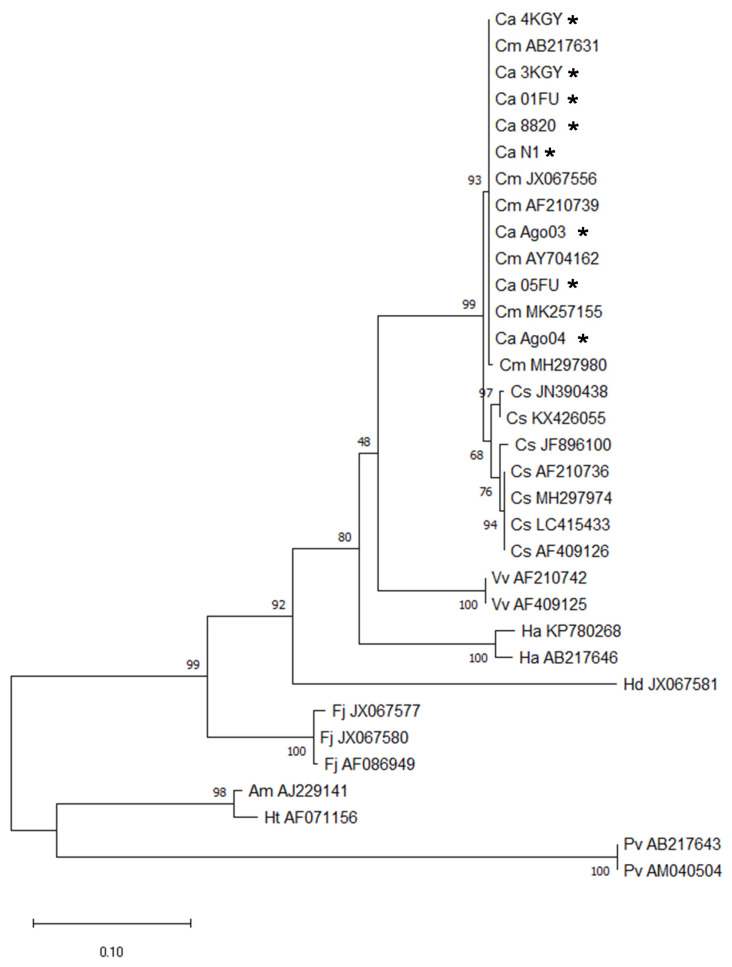
Maximum-likelihood phylogenetic tree from partial sequences of the large subunit (LSU) D1–D2 regions in rDNA of *Chattonella marina* complex strains. The tree was inferred from the K2 + G model. The accession numbers or strain ID used in the present study (asterisks) are shown following the species name. Numbers on the major nodes present maximum-likelihood bootstrap values (1000 replicates). The tree was rooted using *Ascoseira mirabilis*, *Halosiphon tomentosus*, and *Psuedochattonella verruculosa* as the outgroup. Abbreviations of scientific names are as follows: Ca, *Chattonella antiqua*; Cm, *C. marina*; Cs, *C. subsalsa*; Vv, *Vacuolaria virescens*; Ha, *Heterosigma akashiwo*; Hd, *Haramonas dimorpha*; Fj, *Fibrocapsa japonica*; Am, *A. mirabilis*; Ht, *H. tomentosus*; Pv, *P. verruculosa*.

Eight *Chattonella* (Ochrophyta, Raphidophyceae) strains were used in this study. The dates and locations for the isolation of these strains are summarized in Table 1. Two strains were axenic and the others were not. We first confirmed that all strains belonged to *Chattonella* by analyzing sequences of the large subunit (LSU) D1–D2 domain of rDNA (Figure 1) using the method of Shikata et al. [34] and Lum et al. [3]. The strains were sub-cultured in 50-mL Erlenmeyer flasks containing 25 mL of modified SWM-3 medium [35] with a salinity of 32, at 22 or 25 °C, under 400 μmol photons m^−2^ s^−1^ of white fluorescent irradiation on a 14-h:10-h light:dark cycle (light period, 0600 to 2000 local time). The photosynthetic photon flux density (PPFD) in the incubator was measured with a Quantum Scalar Laboratory PPFD sensor (QSL-2101, Biospherical Instruments Inc., San Diego, CA, USA).

### 2.2. Experimental Fish

Cultured red sea bream fingerlings with an approximate total length (TL) of 3 cm and body weight (BW) of 0.5 g were purchased from A-Marine Kindai Co. Ltd. (Wakayama, Japan). The fish were raised for 2–6 months in a 1000-L aquarium with constantly flowing, filtered seawater, and were then acclimatized for a few weeks in 60-L glass aquaria at 25 °C under approximately 5 μmol photons m^−2^ s^−1^ of white fluorescent irradiation on a 14-h:10-h light:dark cycle, during which time they were fed appropriate commercial diets three times per day until the start of the bioassays. The purpose of the long-time raising was that the sensitivity of fish to *Chattonella* is stabilized because hatched fish are unstable physiologically.

Cultured yellowtail fingerlings produced at the Aquaculture Research Department, Fisheries Technology Institute, Japan Fisheries Research and Education Agency (Fukue Island, Japan) were used for experiments. Fish with an approximate TL of 4 cm and BW of 2 g were raised for 2 months in a 500-L aquarium with constantly flowing, filtered seawater, and were then acclimatized for 1–2 in 60-L glass aquaria (600 × 295 × 355 mm^3^) at 22 °C under approximately 5 μmol photons m^−2^ s^−1^ of sunlight pouring through the window (day length: 14–14.5 h), during which time they were fed appropriate commercial diets five times per day until the start of the bioassays.

### 2.3. Toxicity Bioassays

We performed bioassays to quantify the toxicities of the *Chattonella* strains to red sea bream (TL, 11.8 ± 0.3 cm (mean ± SD) and BW, 34.8 ± 2.7 g or TL, 10.3 ± 0.8 cm; BW, 20.7 ± 4.9 g) and yellowtail (TL, 8.2 ± 1.7 cm; BW, 6.1 ± 4.0 g). The bioassays used cultures of *Chattonella* at the late exponential growth phase (approx. 10,000 cells mL^−1^). Cells of strains Ago03 and Ago04 were incubated in 300-mL Erlenmeyer flasks containing 200 mL of modified SWM-3 medium. Cells of the other strains were incubated using the same setup as for subcultures because larger-volume incubations result in unstable growth of these strains, sometimes leading to unexpected death following sedimentation on the bottom of the flasks. For these incubations, the light conditions were 400 μmol photons m^−2^ s^−1^ of white fluorescent irradiation on a 14-h:10-h light:dark cycle (light period, 0600 to 2000 local time) and temperatures were adjusted to 25 °C and 22 °C for bioassays with red sea bream and yellowtail, respectively.

Bioassays were conducted in 13-L glass aquaria (315 × 190 × 230 mm^3^) filled with 6 L of *Chattonella* culture, which were placed in a plastic tray containing temperature-controlled water adjusted to 25 °C (red sea bream) or to 22 °C (yellowtail). The light intensity around the 13-L glass aquaria was approximately 5 µmol photons m^−2^ s^−1^. Aeration in each aquarium was provided using a Pasteur pipette connected to a central air source to generate large bubbles, because red-tide flagellates are sensitive to fine bubbling and turbulence [36]. Three individual red sea breams or five individual yellowtails that had been food-deprived since the previous day were exposed to a *Chattonella* cell suspension or modified SWM-3 medium (control), which was diluted with filtered seawater to adjust the cell density. Time-course changes in fish behavior were then observed for up to 6 h (red sea bream) or 8 h (yellowtail), after which there is usually no more mortality of red sea bream or yellowtail from *Chattonella* exposure. Any fish that were lying on their pectoral fins for more than 5 s (moribund state) were immediately removed from the aquarium because preliminarily observations showed that fish exhibiting this behavior died within 1–2 h, and the presence of dead fish degrades the water quality. Dissolved oxygen (DO) concentrations were maintained at 4–5 mg L^−1^ (low DO condition, red sea bream) or at 8–9 mg L^−1^ (high DO condition, red sea bream and yellowtail) throughout the bioassays by controlling the aeration rate and the number of air outlets. Two DO conditions were used in the bioassays with red sea bream to check for any effects of aeration and high DO on the ranking of ichthyotoxicity in *Chattonella* strains, because aeration and high DO can affect the toxicity of *Chattonella* cells [37]. The bioassay was triplicated.

### 2.4. Cell Size Measurements

Twenty cells of each *Chattonella* strain at the late exponential growth phase were observed under an inverted microscope (Eclipse-Ti, -80i; Nikon Co., Tokyo, Japan) and light micrographs were obtained with a digital camera (DFC290 HD; Leica Microsystems, Wetzlar, Germany). The sizes of cells in the micrographs were measured using Image J software (https://imagej.nih.gov/ij/; achieved on 8 October 2021). We used the software to enclose cells in the images within rectangles to measure maximum Feret’s diameter (cell length) and minimum Feret’s diameter (cell width). Measurements were in triplicate (i.e., *n* = 60 per strain).

### 2.5. O_2_^•−^ Detection

O_2_^•−^ was detected using the chemiluminescence reagent L-012 (FUJIFILM Wako Pure Chemical Corp., Osaka, Japan) [38]. The resultant chemiluminescence was monitored for 30 s using a luminometer (AB-2270 Luminescencer Octa, ATTO Corp., Tokyo, Japan). The O_2_^•−^ levels in cultures were calculated by subtracting the chemiluminescence measured in the presence of 200 U of bovine superoxide dismutase (SOD; FUJIFILM Wako Pure Chemical Corp., Osaka, Japan) from the measured chemiluminescence.

### 2.6. FA Analysis

For lipid extraction, lyophilized cell pellets of *Chattonella* (4.1–9.2 mg) were lysed with an ultrasonic disrupter in 1 mL of hexane (FUJIFILM Wako Pure Chemical Co.), and the resulting homogenate was centrifuged at 2430× *g* at 4 °C for 10 min. The extraction was then repeated two times. Subsequently, the combined extracts were completely evaporated by using a centrifugal evaporator (CVE-2200, Tokyo Rikakikai Co., Ltd., Tokyo, Japan) at 60 °C for 50 min. The dried extracts were then methyl-esterified and purified using a FA methylation kit (Nacalai Tesque, Inc., Kyoto, Japan) and a FA methyl ester purification kit (Nacalai Tesque, Inc.). The purified samples were completely evaporated by using a centrifugal evaporator (CVE- 2200, Tokyo Rikakikai Co., Ltd.) at 60 °C for 50 min. FA methyl esters (FAMEs) were dissolved in 100 μL of acetone and stored at −30 °C until they were analyzed for FA composition.

To determine the FA composition, we performed gas chromatography analysis according to a previous study [39]. The gas chromatography system consisted of a gas chromatograph (GC-14A; Shimadzu Co., Kyoto, Japan), a flame ionization detector, and a capillary column (TC-70, 60 m × 0.25 mm i.d.; GL Science, Tokyo, Japan). The column temperature was programmed for a linear increase of 1 °C min^−1^ from 180 to 230 °C. The temperature of the injection and detector ports was 250 °C. FAMEs were identified on the chromatogram by conventional methods by comparing against the retention time of standards. Supelco 37 Component FAME Mix (Merck KGaA, Darmstadt, Germany) and FAs from Sigma-Aldrich Japan (Merck KGaA, Darmstadt, Germany) were used as the standard for the identification of each FA.

### 2.7. Analysis of Sugar Content and Composition of Monosaccharides

For sugar extraction, cell pellets were lyophilized (FDU-1200, Tokyo Rikakikai Co., Ltd.) and then lysed with an ultrasonic disrupter (UR-21P, Tomy Seiko Co., Ltd., Saitama, Japan) in 1 mL of ultrapure water. The resulting homogenate was centrifuged at 7940× *g* at 4 °C for 10 min. The extraction was then repeated four times. The extracts were transferred into new conical tubes containing 40 mL of 99.5% ethanol (FUJIFILM Wako Pure Chemical Co.) and kept at −4 °C for at least 10 h, after which the mixtures were centrifuged at 2150× *g* at 4 °C for 20 min, followed by two more re-suspensions and centrifugations in ethanol. The resulting sugar pellet was dried at room temperature and dissolved in 5 mL of ultrapure water by vortex mixing and sonication. The total sugar in 5 mL of each extract was determined by the modified phenol–sulfuric acid method [40]. Light absorbance at 480 nm was determined using a microplate reader (Multiskan GO; Thermo Fisher Scientific Co., Ltd., Waltham, MA, USA). D-glucose (D-Glc; FUJIFILM Wako Pure Chemical Co.) was used as a standard for the determination of total sugar.

To analyze sugar composition, the cell pellets were first hydrolyzed into monosaccharides with 2 M trifluoroacetic acid at 120 °C for 1 h. After the removal of trifluoroacetic acid with a SpeedVac (Thermo Fisher Scientific Co., Ltd.), the sugar composition was determined by high-performance anion-exchange chromatography with pulsed amperometric detection (HPAEC-PAD) using a Dionex ICS-5000+ liquid chromatograph equipped with a CarboPac PA-1 column and a pulsed amperometric detector (Thermo Fisher Scientific Co., Ltd.), as described previously [41].

### 2.8. Statistical Analyses

The data were tested for homogeneity of variances using Levene’s test. Where the variances were homogeneous, we used one-way analysis of variance and multiple comparisons with Tukey’s honestly significant difference (HSD) test to test for differences in *Chattonella* biological parameters among strains. Data not showing homogeneous variances were log-transformed, and a Levene’s test was performed once again. When the variances were not homogeneous even after log-transformation, we used the Dunnet T3 test.

To explore the parameters influencing the ichthyotoxicity of *Chattonella*, we calculated the Spearman’s rank correlation coefficients (*ρ*) between moribundity rate and *Chattonella* parameters of cell density, cell size, O_2_^•−^ level, and contents of sugar and each fatty acid.

All analyses were performed with IBM SPSS Statistics Desktop Version 19.0 for Windows (IBM Japan, Tokyo, Japan) using a significance level of *p* < 0.05.

## 3. Results

### 3.1. Ichthyotoxicity

**Figure 2:**
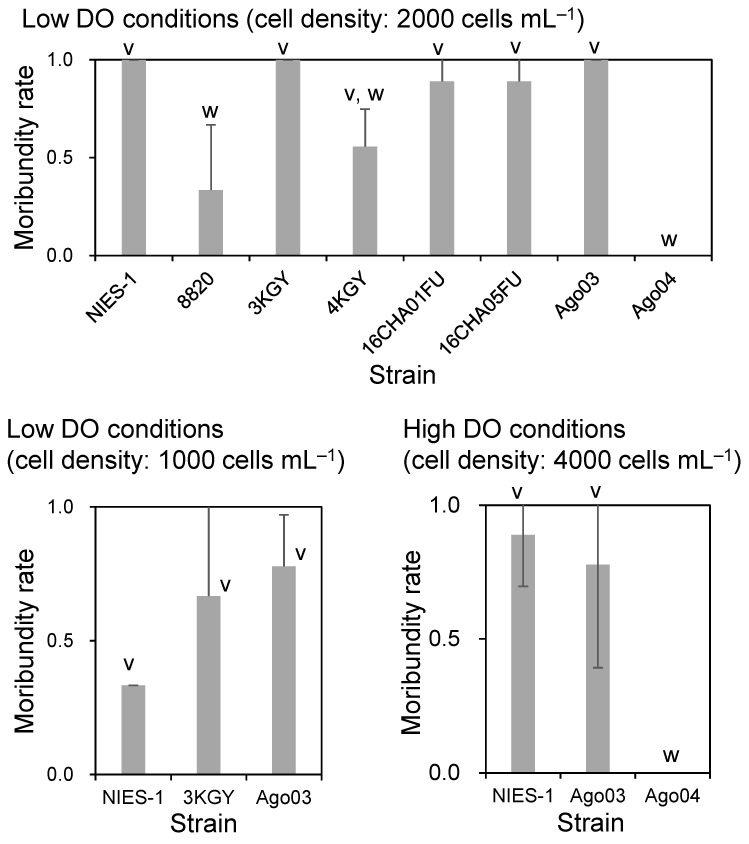
Toxicity of *Chattonella* strains to red seabream under different dissolved oxygen concentrations and exposure cell densities. Each bar represents the mean ± SD of triplicate measurements. Means that are not significantly different are labeled with the same letter (v or w; *p* < 0.05).

We first conducted bioassays under low DO conditions (4–5 mg L^−1^) using red sea breams of TL 11.8 ± 0.3 cm (mean ± SD) and BW 34.8 ± 2.7 g. There were significant differences in average moribundity rates of red sea bream among *Chattonella* strains at an exposure density of 2000 cells mL^−1^ (Figure 2). Multiple comparison analysis separated the strains into three groups by average moribundity rate: highly toxic strains (NIES-1, 3KGY, 16CHA01FU, 16CHA05FU, and Ago03), an intermediately toxic strain (4KGY), and low-toxicity strains (8820 and Ago04). For three of the highly toxic strains—NIES-1, 3KGY, and Ago03, which brought all red sea breams to a moribund state at 2000 cells mL^−1^—we conducted an additional bioassay at half the cell density. The average moribundity rates ranged from 0.33 to 0.78, but there was no significant difference among strains (Figure 2).

For two of the strains that were highly toxic under low DO conditions, NIES-1 and Ago03, and one that showed low toxicity, Ago04, we conducted the bioassay under high DO conditions (8–9 mg L^−1^) with an exposure density of 4000 cells mL^−1^ using red sea breams (TL, 10.3 ± 0.8 cm; BW, 20.7 ± 4.9 g). Most fish exposed to the highly toxic strains became moribund, but all fish exposed to the low-toxicity strain Ago04 remained normal until the end of the experiment (6 h) (Figure 2).

**Figure 3:**
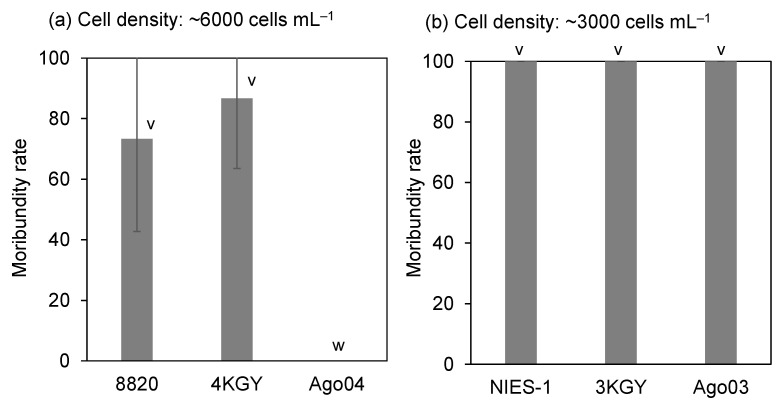
Toxicity of *Chattonella* strains to yellowtail at different cell densities. Each bar represents the mean ± SD of triplicate measurements. Low-toxicity strains (8820, 4KGY, Ago04) (**a**) and highly toxic strains (NIES-1, 3KGY, Ago03) (**b**) were exposed to fish at cell densities of ~6000 and ~3000 cells mL^−1^, respectively. Means that are not significantly different are labeled with the same letter (v or w; *p* < 0.05).

When yellowtails (TL, 8.2 ± 1.7 cm; BW, 6.1 ± 4.0 g) were exposed to dense cultures (around 6000 cells mL^−1^) of strains showing low or intermediate toxicity to red sea breams, some (strains 8820 and 4KGY) or all (Ago04) fish remained normal until the end of the experiment (8 h). There were significant differences between the moribundity rates of yellowtails exposed to strain 8820 or 4KGY and strain Ago04 (Figure 3). For the strains highly toxic to red sea breams (NIES-1, 3KGY, and Ago03), yellowtails were exposed to dilute cultures (around 3000 cells mL^−1^). All yellowtails became moribund within 8 h (Figure 3). None of the red sea breams or yellowtails displayed abnormal behavior within the 6-h or 8-h control exposure (modified SWM-3 medium).

### 3.2. Cell Size

**Figure 4:**
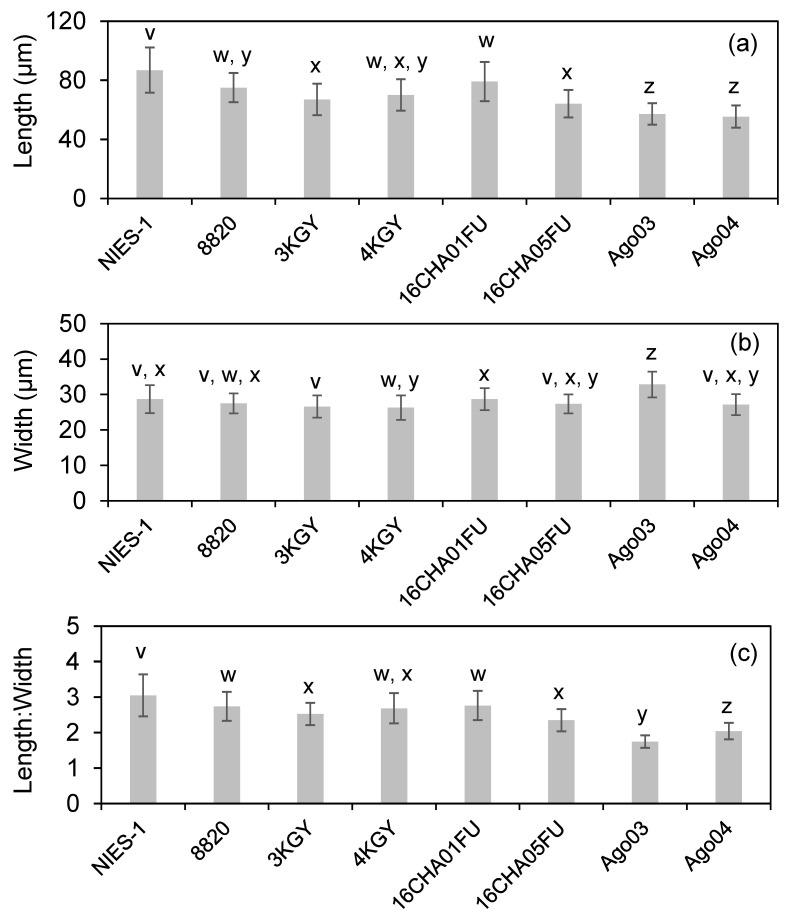
Length (**a**) and width (**b**) of cells and the length:width ratio (**c**) of *Chattonella* strains (*n* = 60). Each bar represents the mean ± SD of triplicate measurements. Means that are not significantly different are labeled with the same letter (v–z; *p* < 0.05).

We measured the cell sizes of eight *Chattonella* strains at the late exponential growth phase. The average cell length, width, and length:width ratio ranged from 55 to 87 µm, from 26 to 33 µm, and from 1.7 to 3.1, respectively (Figure 4). Cell lengths were categorized into three groups by multiple comparison analysis: a long strain (NIES-1), short strains (Ago03 and Ago04), and five intermediate strains (the others) (Figure 4a). For cell width, Ago03 was the largest and the others were similar (Figure 4b). The length:width ratio was categorized into four groups: a high-ratio strain (NIES-1), a lowest-ratio strain (Ago03), a lower-ratio strain (Ago04), and five intermediate-ratio strains (the others) (Figure 4c). However, correlation analyses showed that neither the cell length (*ρ* = 0.22, *p* = 0.44), width (*ρ* = 0.60, *p* = 0.27), nor the length:width ratio (*ρ* = 0.07, *p* = 0.86) were significantly correlated with moribundity rates of red sea bream exposed to *Chattonella* strains at 2000 cells mL^−1^ under low DO conditions.

### 3.3. O_2_^•−^ Production

**Figure 5:**
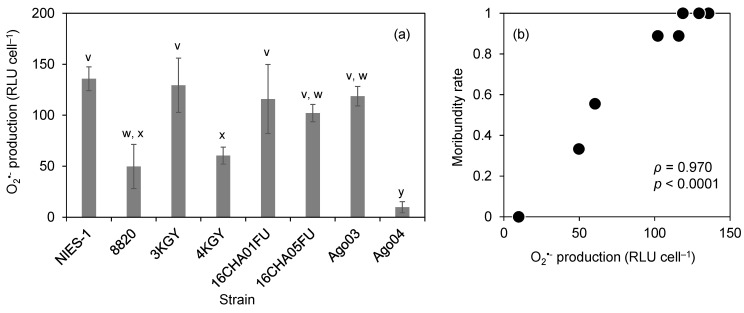
Superoxide (O_2_^•−^) production levels of *Chattonella* strains (**a**) and their relationship with the moribundity rate shown in Figure 2. Data of control (no *Chattonella* cell) are also plotted (**b**). Each bar represents the mean ± SD of triplicate measurements. Means that are not significantly different are labeled with the same letter (v–y; *p* < 0.05). RLU, relative luminescence units.

We measured the cellular O_2_^•−^ production levels of eight strains in the late exponential growth phase (approx. 10,000 cells mL^−1^). The average O_2_^•−^ production ranged from 10 to 136 relative luminescence units (RLU) cell^−1^ (Figure 5a). The multiple comparison analysis of O_2_^•−^ production separated the strains into three groups: a low-production strain (Ago04), two intermediate-production strains (8820 and 4KGY), and five high-production strains (the others). The O_2_^•−^ production levels of strains were significantly correlated with moribundity rates of red sea bream exposed at 2000 cells mL^−1^ under low DO conditions (Figure 5b; *ρ* = 0.97, *p* < 0.0001).

**Figure 6:**
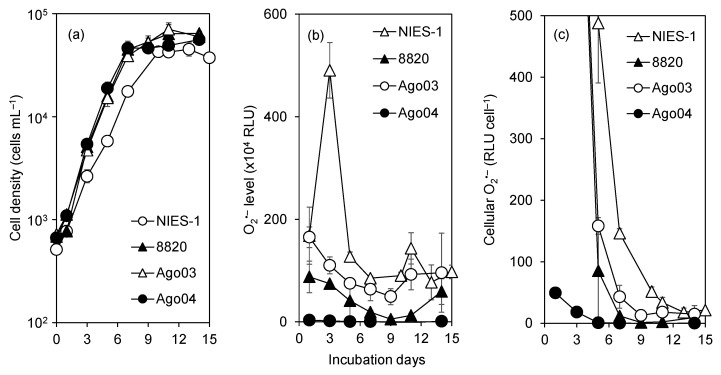
Time-course variations in cell density (**a**), superoxide (O_2_^•−^) level in culture (**b**), and cellular O_2_^•−^ production level (**c**) in two highly toxic (Ago03 and NIES-1) and two low-toxicity (Ago04 and 8820) *Chattonella* strains. Data points in each plot represent the mean ± SD of triplicate measurements. RLU, relative luminescence units. This experiment was conducted in the absence of fish.

We monitored time-course variations in cell density and O_2_^•−^ levels for 15 d in two highly toxic strains, Ago03 and NIES-1, and two low-toxicity strains, Ago04 and 8820. This experiment was conducted in the absence of fish. Cell density increased exponentially until day 7 or 10 for all four strains (Figure 6a). The patterns of variation of total O_2_^•−^ (Figure 6b) and cellular O_2_^•−^ production (Figure 6c) were similar among all strains; maximum values were recorded on day 1 or 3, after which the values declined until day 5–9. Total O_2_^•−^ levels and cellular O_2_^•−^ production levels were higher in highly toxic strains than in low-toxicity strains throughout the experimental period.

**Figure 7:**
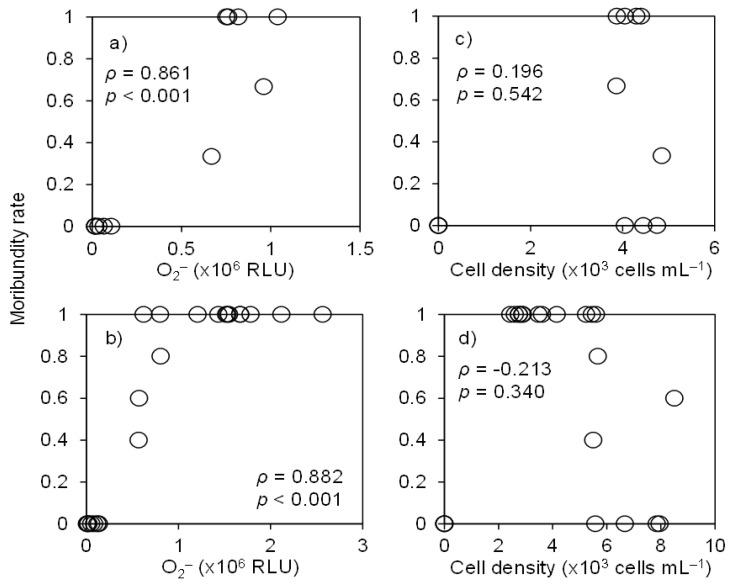
Relationship between superoxide level (**a**,**b**) and cell density (**c**,**d**) of *Chattonella* cultures, and toxicity to red sea bream (**a**,**c**) and yellowtail (**b**,**d**) in the bioassays shown in Figure 2 (high DO conditions) and Figure 3. RLU, relative luminescence units. Data of control (no *Chattonella* cell) are also included.

We measured the O_2_^•−^ level of the cultures at the start time of the bioassays with red sea bream and yellowtail under high DO conditions. The O_2_^•−^ levels of the strains and the control (culture medium diluted with filtered seawater) ranged from 0.03 to 2.6 × 10^6^ RLU and from 0.005 to 0.02 × 10^6^ RLU, respectively. Correlation analysis revealed significant correlations between the O_2_^•−^ level and moribundity rates of both red sea bream and yellowtail (Figure 7a,b; *ρ* > 0.8, *p* < 0.001), whereas there was no significant correlation between cell density and moribundity rates (Figure 7c, d; *ρ* < 0.3, *p* > 0.3).

### 3.4. Fatty Acid Composition

**Figure 8:**
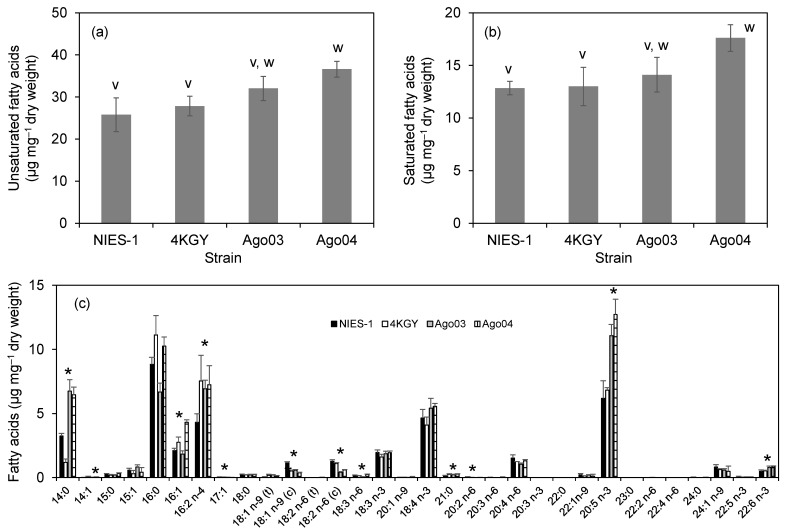
Content of unsaturated fatty acids (**a**) and saturated fatty acids (**b**) and their composition (**c**) in two highly toxic (NIES-1, Ago03) and two low-toxicity strains (4KGY, Ago04) of *Chattonella*. Each bar represents the mean ± SD of triplicate measurements. Means that are not significantly different are labeled with the same letter (v or w; *p* < 0.05). Fatty acids in (**c**) labeled with an asterisk had significantly different contents among the strains.

We analyzed the FA content of two highly toxic strains (NIES-1 and Ago03) and two low-toxicity strains (4KGY and Ago04). The unsaturated fatty acid (UFA) content ranged from 26 to 37 µg mg^−1^ dry weight (Figure 8a). The UFA content of the least toxic strain, Ago04, was significantly higher than those of NIES-1 and 4KGY. The saturated fatty acid (SFA) content ranged from 13 to 18 µg mg^−1^ dry weight (Figure 8b). The SFA of Ago04 was significantly higher than that of NIES-1. Total FA content ranged from 39 to 54 µg mg^−1^ dry weight. The total FA content of Ago04 was significantly higher than those of NIES-1 and 4KGY. The most abundant FA in NIES-1 and 4KGY was 16:0 (palmitic acid), and that in Ago03 and Ago04 was 20:5 n-3 (eicosapentaenoic acid (EPA)) (Figure 8c). The contents of 13 FAs differed significantly among *Chattonella* strains. However, correlation analysis showed that no FA was significantly correlated with the moribundity rate of red sea bream exposed to strains at 2000 cells mL^−1^ under low DO conditions (*ρ* = −0.95 to 0.95, *p* = 0.05 to 0.89).

### 3.5. Sugar Content and Composition of Monosaccharides

**Figure 9:**
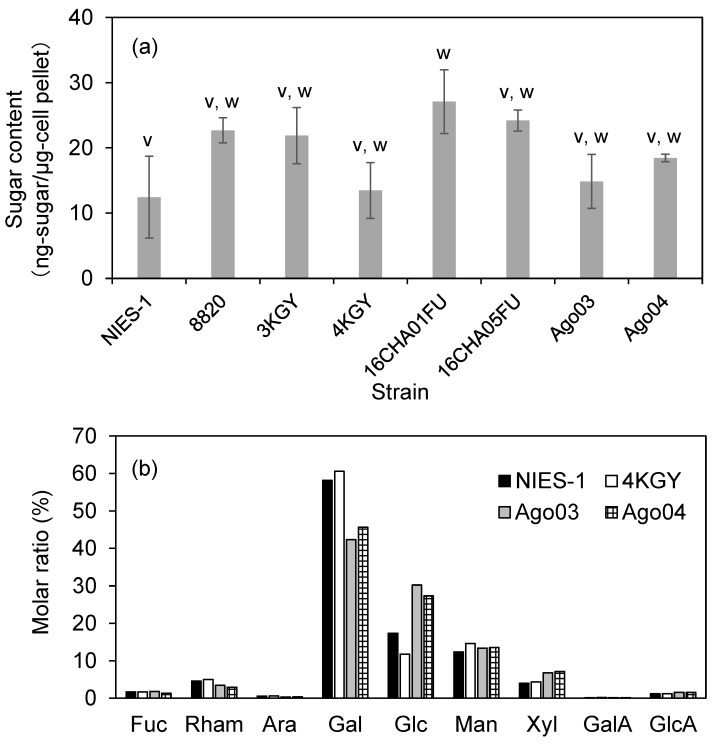
Sugar content (**a**) and composition of monosaccharides (**b**) of *Chattonella* strains. Each bar represents the mean ± SD of triplicate measurements. Means that are not significantly different are labeled with the same letter (v or w; *p* < 0.05). Abbreviations of sugars are as follows: Fuc, fucose; Rham, rhamnose; Ara, arabinose; Gal, galactose; Glc, glucose; Man, mannose; Xyl, xylose; GalA, galacturonic acid; GlcA, glucuronic acid.

We analyzed the sugar contents of all eight strains at the late exponential growth phase. Cell pellets were collected from the same cultures used for the O_2_^•−^ measurements shown in Figure 5a. The average sugar content ranged from 12 to 27 ng-sugar µg-cell pellet^−1^ (Figure 9a). Although sugar content differed significantly among strains (Figure 9a), correlation analysis showed that sugar content was not correlated with moribundity rates of red sea bream exposed to strains at 2000 cells mL^−1^ under low DO conditions (ρ = −0.23, *p* = 0.58).

We determined the sugar composition of two highly toxic strains (NIES-1 and Ago03) and two low-toxicity strains (4KGY and Ago04) (n = 2). The composition of monosaccharides showed enrichment in galactose (Gal, 40–60 mol%) and glucose (Glc, 10–30 mol%) in all strains (Figure 9b). However, the relative contents of Gal and Glc were clearly higher and lower, respectively, in NIES-1 and 4KGY than in Ago03 and Ago04.

## 4. Discussion

There were large differences in toxicity to red sea bream and yellowtail among *Chattonella* strains tested in this study. Although fish body sizes and cell exposure densities were not exactly the same, the ranking of toxicity scarcely differed among DO conditions and fish species (Figure 2 and Figure 3). These findings indicate that ichthyotoxicity in *Chattonella* is a property inherent to each strain.

One important finding about differences in toxicity among strains in the present study is that a non-toxic strain (Ago04) was found. At first, we suspected that this strain was not *Chattonella*, but this doubt was removed following a phylogenic analysis. It is interesting that strain Ago03 isolated from the same seawater sample as Ago04 was highly toxic. Similarly, 4KGY (low toxicity) and 3KGY (highly toxic) came from the same seawater source. These results indicate that strains with different toxicities coexist in natural seawater. Moreover, even NIES-1, isolated over 40 years ago, had high toxicity, as high as younger strains such as 3KGY and Ago03, indicating that *Chattonella* can maintain ichthyotoxicity for a long period in culture.

Although there is some ambiguity in morphological criteria for distinguishing species of *Chattonella* [8], *Chattonella* is currently identified into three species (*C. antiqua*, *C. marina*, and *C. ovata*) in red-tide monitoring, at least in Japan. We therefore investigated the relationship between ichthyotoxicity and morphological features in the strains. There were significant differences in morphological characteristics among the eight strains at the same growth phase. According to Hara [4], the three species of *Chattonella* can be distinguished by their main characteristics of cell size, the presence or absence of a slender posterior tail, and developed vacuoles in the ectoplasm. At first, we did not observe developed vacuoles in the ectoplasm in all strains, indicating that *C. ovata* might not have been included in the strains. The strains were generally separated into three groups by cell length in this study, but the average cell lengths were all greater than about 50 µm, which is a criterion that distinguishes *C. antiqua* from *C. marina* [4]. However, Ago03 and Ago04 lacked the slender posterior tail and their length:width ratios were much lower than in the other strains, indicating that these two strains may be *C. marina*. Interestingly, the monosaccharide composition clearly separated two groups: *C. marina*-like strains (Ago03 and Ago04) and *C. antiqua*-like strains (NIES-1 and 4KGY). However, there was no clear difference in ichthyotoxicity between *C. antiqua*-like and *C. marina*-like strains because there was no significant difference between ichthyotoxicity based on cell size or between ichthyotoxicity of the *C. marina*-like strain Ago03 and the *C. antiqua*-like strains.

We demonstrated that the O_2_^•−^ level is correlated with ichthyotoxicity over multiple *Chattonella* strains. Ishimatsu et al. [12] similarly reported that the O_2_^•−^ production level corresponds to ichthyotoxicity, although they could not determine any significant differences between strains because they only compared two strains. There are arguments for and against the involvement of ROS in the ichthyotoxicity of *Chattonella*. Artificial O_2_^•−^ produced by xanthin oxidase does not kill damselfish at the production level of *Chattonella* cells [23]. Similarly, when goldlined seabream was exposed to hydrogen peroxide at concentrations 25 times the lethal level from *Chattonella* cells, the branchial dysfunctions observed in the *Chattonella* exposure scarcely occurred and the median lethal time was double that in the *Chattonella* exposure [42]. However, the glycocalyx in a *Chattonella* cell contains NADPH oxidase [43], so *Chattonella* cells can stick firmly to branchial epithelia and produce O_2_^•−^ there. Moreover, it is known that branchial mucus promotes drastic O_2_^•−^ production by *Chattonella* [44]. Ishimatsu et al. [12] reported that yellowtail had higher mortality in the light than in the dark; the extracellular secretion of superoxide controlled by photosynthesis was suppressed in the dark treatment [27,45]. These observations combined suggest that dissolved O_2_^•−^ from motile *Chattonella* cells alone cannot kill fish, but it is possible that O_2_^•−^ from *Chattonella* cells attached to gills is one of the main chemicals responsible for the dysfunction of fish gills; the present study supports this possibility.

It has been suggested that dominant unsaturated fatty acids such as EPA display toxicity to fish, and the toxicity increases drastically with coexisting ROS [23]. However, the stable chemical properties of FAs and lipoperoxides do not support the observation that the ichthyotoxicity of *Chattonella* is extremely unstable. Moreover, FAs exist largely inside cells in a healthy algal culture [46], which is inconsistent with the observation that exposure to ruptured *Chattonella* cells does not kill whole fish. The FA compositions of *Chattonella* strains in the present study were similar to those found in previous studies [45,47], and EPA was one of the dominant fatty acids. Although there were significant differences among strains in the contents of several fatty acids including EPA, the differences did not correspond with levels of ichthyotoxicity. EPA content was highest in the non-toxic strain Ago04. To our knowledge, there is no study that can provide a relationship between FAs and ichthyotoxicity in *Chattonella* strains with a quantitative underpinning.

In comparison with FA composition and content [14], the O_2_^•−^ production level fluctuates widely under various environmental conditions and growth phases [27,38,48]. Even if synergism between FAs and O_2_^•−^ is the driver of ichthyotoxicity, the O_2_^•−^ level would have a far greater impact on the degree of ichthyotoxicity than FA composition and content.

Sugar can play an important role in the mechanism by which *Chattonella* cells attach to gill tissue [32]. However, we found no correspondence between total sugar content and the composition of monosaccharides and ichthyotoxicity. It has been experimentally demonstrated that *Chattonella* cells are more prone to attach to fish gills than other phytoplankters [31], but the mechanism is poorly understood. The effects of molecular weight and higher-order structure on the viscosity of polysaccharides including glycocalyx should be investigated in future studies.

## 5. Conclusions

It is necessary to determine the chemical(s) responsible for gill dysfunction to elucidate the mechanism of aquacultured fish mortality by *Chattonella*. Previous studies have suggested multiple candidates for the chemical but have not achieved identification. It is possible that using animals or cells other than whole fish in toxic bioassays involves complex phenomena. In the present study, we performed bioassays using whole aquacultured fishes and thereby showed that O_2_^•−^ production quantitatively correlates with the mortality of aquacultured fish. On the other hand, the content and composition of FAs including EPA, which had been suspected as a toxic factor, did not correlate with ichthyotoxicity. The role and mechanism of O_2_^•−^ in the process of gill dysfunction remain to be determined in the future.

## Figures and Tables

**Table 1:** *Chattonella* strains isolated from seawater around Japan.

Strain Name	Date Collected	Location	Contamination Status
NIES-1	September 1978	Harima-Nada	Axenic
8820	20 August 2008	Yatsushiro Sea	Xenic
3KGY	3 June 2010	Yatsushiro Sea	Xenic
4KGY	3 June 2010	Yatsushiro Sea	Axenic
16CHA01FU	6 July 2016	Seto Inland Sea	Xenic
16CHA05FU	6 July 2016	Seto Inland Sea	Xenic
Ago03	9 July 2013	Ago Bay	Xenic
Ago04	9 July 2013	Ago Bay	Xenic

## Data Availability

Data are available within the article.
